# Development and Validation of an Online Program for Promoting Self-Management among Korean Patients with Chronic Hepatitis B

**DOI:** 10.1155/2013/702079

**Published:** 2013-01-15

**Authors:** Jinhyang Yang

**Affiliations:** Department of Nursing, College of Medicine, Inje University, 633-165 Gaegum-dong, Busanjin-gu, Busan 614-735, Republic of Korea

## Abstract

The hepatitis B virus is second only to tobacco as a known human carcinogen. However, chronic hepatitis B usually does not produce symptoms and people feel healthy even in the early stages of live cancer. Therefore, chronically infected people should perceive it as a serious health problem and move on to appropriate health behaviour. The purpose of this paper is to develop and validate an online program for promoting self-management among Korean patients with chronic hepatitis B. The online program was developed using a prototyping approach and system developing life cycle method, evaluated by users for their satisfaction with the website and experts for the quality of the site. To evaluate the application of the online program, knowledge and self-management compliance of the subjects were measured and compared before and after the application of the online program. There were statistically significant increases in knowledge and self-management compliance in the user group. An online program with high accessibility and applicability including information, motivation, and behavior skill factors can promote self-management of the patient with chronic hepatitis B. Findings from this study allow Korean patients with chronic hepatitis B to engage in proactive and effective health management in the community or clinical practice.

## 1. Introduction

The hepatitis B virus is known as not only the second highest carcinogen after smoking, but also the cause of 75% of primary hepatocellular carcinoma [1]. A carrier of hepatitis B is 30–100 times more likely to have a risk of dying from liver disease than the average person [2]. Approximately 350 million people have hepatitis B worldwide, and, every year, about 620,000 die of liver diseases associated with hepatitis B [3, 4]. Hepatitis B is a disease that is more common in Asians than Westerners. Asian Americans have an infection rate of 5–15%, which is, approximately, more than 20 times higher than the hepatitis B infection rate of the entire population of the US [5]. Korea was ranked 5th in cancer incidence rate in 2010 for liver cancer, and is ranked second in cancer mortality [6]. The prevalence of hepatitis B in Korea was 4.0% of the population over the age of 30 [7]. Likewise, even today, hepatitis B remains a common disease among the average person, but there is a lack of management on disease monitoring and treatment compliance of hepatitis B virus carriers or patients [8]. 

Currently, a significant number of hepatitis B patients in Korea are caused by vertical infection from the mother during the perinatal period. Due to the fact that these patients live several years unaware that they are infected, several generations of a family may be affected by hepatitis B, and when they reach the age of 40–50, it is highly likely to progress to liver cirrhosis or liver cancer [9]. Most patients with chronic hepatitis B do not have any specific symptoms until they develop complication and then go through the long-term disease progression period that takes about 10–30 years. It usually goes through the process of immune tolerance, immune clearance, nonreplicative, and reactivation phase. Generally, the targets of treatment are patients with HBeAg-positive infection in immune clearance phase and others with HBeAg-negative infection in reactivation phase. The targets of regular disease monitoring that do not need to receive immediate treatment but must prepare for immune clearance phase or reactivation phase are hepatitis B virus carriers in the immune clearance and nonreplicative phase [2]. Therefore, there is a need to raise the regular disease monitoring and treatment compliance along with daily life management based on the disease progress and accurate disease knowledge with regard to chronic hepatitis B patients. 

According to precedent studies, it was reported that hepatitis B patients have a high level of noncompliance to the regular health checkups or prescriptions [10], have a low quality of life related to health [11], and have a low level of knowledge on disease and disease management, giving rise to deterioration of personal relations or even psychiatric issues such as fear, depression, and anxiety [12]. Furthermore, according to the qualitative research investigating patterns of health care behavior among patients with chronic hepatitis B [13], hepatitis B virus carriers had a low level of disease monitoring through regular medical checkups, and the extent of treatment compliance varied by the pattern of health care in the case of patients subject to treatment. In particular, the treatment compliance in task-oriented type in daily life and body-oriented healthcare type were very low. In addition, most of the hepatitis B patients are not aware of their disease and feel that they are healthy due to the nonvisible and nonspecific characteristics of hepatitis B. Hence, there is a risk of unconsciously spreading the virus to other people [9]. 

When considering the chronic progress characteristics throughout several decades of hepatitis B, the promotion of patient's self-management ability of chronic hepatitis B is very important. The theoretical frameworks for a program to promote self-management ability of the patients with chronic diseases include knowledge, motivation, and behavior skill factors of disease control [14, 15]. The misunderstanding of hepatitis B patients' regard of the disease often leads to their indifference or inappropriate health behavior [16]. Therefore, it is necessary for health care providers to integrate the existing health care information on hepatitis B to provide accurate knowledge about the health care management of the disease. The motivation factor can be explained as the self-efficacy required for remembering and managing the need and content of disease control or treatment, even if the patient has a good knowledge of it. Behavior skill factors refer to the establishment of action plans and psychosocial management for disease monitoring, treatment compliance, and maintenance of a healthy lifestyle. The patient with chronic hepatitis B should ultimately foster appropriate self-management ability by acquiring accurate and systematic knowledge suitable to oneself, developing motivation through increasing self-efficacy, and pursuing behavioral change through learned skills. The self-management ability has helped patients with chronic disease bring about a result of maintaining and promoting health from the aspect of preventing severe disease and lifelong health management [14]. 

There must be a premise of an understanding on the fundamental basis of the chronic hepatitis B patients' health behavior to promote their self-management, and health management suitable for an individual's disease progress state, health issues, and health needs must be carried out based on that premise [7]. However, existing studies usually focus on the treatment of chronic hepatitis B itself [17] and mostly consist of survey on knowledge level [18], survey on infection rate and vaccination rate [19], studies on the correlation between some variables [20], and studies on prevention programs [21]. It is difficult to find studies that have developed and applied a self-care promotion program in consideration of multidimensional factors so that lifelong health management can be internalized. For patients with hepatitis B who have disease characteristics of nonvisible, nonspecific, relatand chronic progression, it is viewed that fostering self-management abilities will be effective by acquiring the most suitable information according to a patient's disease progress state and health care behavior through an online program, and by enhancing self-efficacy and the ability to cope through promoting interactions for problem-solving between experts or other patients. Korean patients with chronic disease can easily access online resources for self-management of their own disease due to South Korea's high-speed wireless Internet penetration rate, which is marked at 100 percent [22]. 

The online program is a system which allows the patient to systematically receive required information with the most updated content through a personal computer without limitation of time and location, to consult one's disease-related health issues with experts, and to be able to foster one's health care ability through various multimedia learning. However, the existing Internet information system mainly consists of content focusing on unidirectional knowledge delivery on the prevention and treatment of hepatitis B and is evaluated as having a lack of utility since it is unable to reflect the reliability assessment of information and user needs [23]. Rather than delivering text-oriented textbook-like content to patients with hepatitis B in various disease progress states, the use of an online program for lifelong health management would be an effective system to consider, which would include an approach of fostering patient's self-management capacities in patient health care by utilizing multimedia and a selective path that is most suitable for disease control, health issues, and health needs of oneself, including one's family. 

An online program to promote self-management capacities in patients with chronic hepatitis B can be used to increase the knowledge level on the disease's management and to promote the compliance to overall self-management through enhancing the motivation and behavior skill factors of the patients with chronic hepatitis B. The levels of knowledge and self-management compliance in this study will be measured through self-reporting questionnaires. The compliance to self-management will allow patients to avoid serious consequences such as liver cirrhosis or liver cancer in advance and furthermore will be helpful for the patients to establish a healthy lifestyle from the aspect of lifelong health management. Therefore, the purpose of this study is to develop an online program for patients with chronic hepatitis B to enhance their self-management capacities of the disease, and to evaluate its application. 

## 2. Materials and Methods

**Figure 1:**
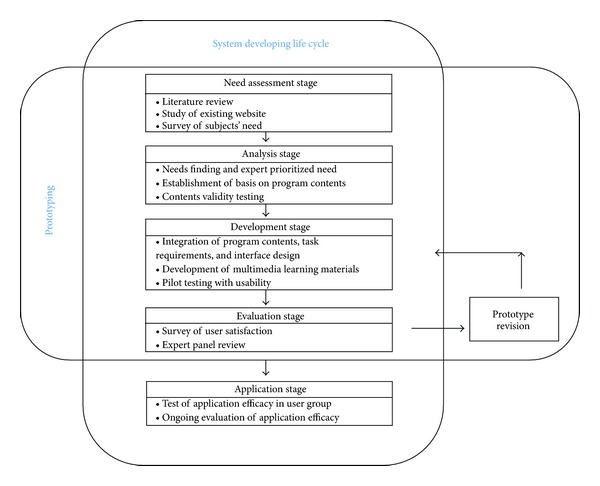
Stages for the development of an online program for promoting self-management.

A design with a combination of software prototyping approach and system develop life cycle [24] was applied to develop an online program for self-management in patients with chronic hepatitis B (Figure 1). After first identifying the user requirements through this design, a prototype online program for promoting self-management was developed and revised by receiving feedback from users and experts. By repeating this process, the image of the actual program was established, its webpage was designed and the program was developed using the traditional system developing life cycle. In this study, the users are those patients who visit a university hospital in which the author is affiliated, who understand the importance of the present study, and who also provided a signed written consent form for their study participation. Since the patient recruitments were made in three different survey periods (need assessment stage, evaluation stage, and application stage), patient participation in each survey period was different from one another. The expert panel was formed by the people who work at the university hospitals where the author is affiliated, and the panel suggests a group people who specialize in treatment and disease management of hepatitis B, including medical doctors, nurses, and online health program coordinators. All of them were introduced to the present study at an early stage of the study through emails and also signed up for the multiple stage study participation. To protect the rights of those who participated in this study, ethical approval of the study was made by the institutional review board of the university hospital in which the author is affiliated.

### 2.1. Need Assessment Stage

The major issues and health issues related to the management of chronic hepatitis B were identified through literature review, websites, patients, and their families. Initially, the contents that are related to self-management of the chronic hepatitis B disease were analyzed by reviewing literature and websites to form questionnaire issues. Through consultations with an expert panel, a questionnaire including 25 items was established. Author and research assistants of this study requested patient participation in the questionnaire survey through face-to-face advertisement of the study to the hospital-visiting chronic hepatitis B outpatients. By recruiting 52 chronic hepatitis B patients and 15 family members of the patients (who signed the written consent form), the program requirements and Internet utilization characteristics were investigated. Also, additional interviews were conducted to confirm the unrecognized or misinformation on the management of chronic hepatitis B.

### 2.2. Analysis Stage

To analyze the data collected in the first stage, an expert panel was formed consisting of two physicians, three nurses, and one online health program coordinator, all of whom are involved in the treatment or management of chronic hepatitis B. First of all, major issues and health problems associated with lifelong health management of patients with chronic hepatitis B were uncovered, their priority was then set, and strategies for solving the problems were sought out. Discrepancies were discussed and a consensus was reached. Furthermore, each item related to the program was verified for its content validity by eight other experts among the expert panel pool in this study. Each item was graded on a 4-point scale, where 4 points equate to “Strongly agree” and 1 point equates to “Strongly disagree,” and the issues with a minimum content validity index (CVI) of 0.80 were selected. Based upon the analysis results, the foundation of the program contents was established.

### 2.3. Development Stage

Through this basic process, an online program for lifelong health management with high accessibility and applicability was constructed for promoting self-management of the patient with chronic hepatitis B. This online program broadly consists of 6 main menus: Introduction to Lifelong Management of hepatitis B, What is hepatitis B?, Lifelong Health Management, Management of My Liver Health, Bulletin Board, and Sharing Board, with 24 submenus under them. Apart from this, three menus with much importance and applicability were extracted separately and set vertically on the right hand screen. If the main menu is connected to the information factor, motivation factor, and behavior skill factor, which are the factors to promote self-management in chronic disease patients, the menu “Introduction to Lifelong Management of hepatitis B” was designed as a foundation for strengthening these three factors. The menu, “What is hepatitis B?” is a stage for strengthening disease-related knowledge, which is an information factor. To enhance the learning effect, various audiovisual materials such as PowerPoint presentations, photos, and videos were utilized. The menu “Lifelong Health Management” strengthens the behavior skill factors by assisting in setting specific goals and establishing an action plan. The menu “Management of My Liver Health” strengthens the behavior skill factor by helping the patient understand and implement disease control through pop-up windows or alert messages, as well as individual algorithms. The menu “Bulletin Board” was made to enable viewers to get the latest information. Finally, in “Sharing Board,” motivation factors were strengthened through one-on-one consulting with experts and experience sharing with other patients. 

Submenus were completed after going through discussions on whether to use images and videos, ways of expressing content, and navigation strategies depending on the topic and content. The developed online program is the uppermost system, which is the initial screen, and is registered at the web address: http://www.hepb.co.kr/. 

### 2.4. Evaluation Stage

The developed online program conducted the evaluation on user satisfaction and expert panel review with a survey. The evaluation on user satisfaction used a tool developed by Park et al. [25]. This tool consists of six questions related to the system (system efficiency, system convenience, design), and 11 questions related to content (content relevancy and content usefulness). Each question is rated on a 5-point scale, with a higher score meaning higher satisfaction. Data collection was carried out with a mail survey, which was enclosed with a questionnaire on 28 outpatients with chronic hepatitis B at a university hospital for approximately four weeks. Participant recruitment was conducted by posting the evaluation participation on the hospital outpatient bulletin board and through face-to-face advertisement of the participation to the hospital-visiting chronic hepatitis B outpatients. Author and research assistant initially explained the method of online program evaluation by phone or by face-to-face meeting to those patients who orally agreed to study participation and sent them written consent forms and questionnaires. After that, the corresponding website address was given and patients were asked to mail their written consent form and the questionnaire to the author after evaluating the program at home using their own computers. 

The evaluation of the online program by experts used the health care website evaluation tool developed by Chung & Park [26]. This tool is a 27-question survey consisting of eight areas: purpose, appropriateness, accuracy, credibility, ease of use, interactivity, currency, and authority. Each question is rated on a 5-point scale, with a higher score meaning higher satisfaction. Data collection was carried out by a survey via e-mail attached with a questionnaire by a total of 20 experts consisting of three physicians, 14 nurses, and three online health program coordinators for about four weeks. The author introduced the program evaluation method to those on the expert panel through email and requested response to the questionnaire. 

The questionnaire evaluation by users and experts was carried out after receiving written consent on participation in the study after the author and research assistants explained the purpose and method of the study, the spontaneity of participation and withdrawal, and the content of the questionnaire to the subjects. The current online program for promoting self-management was completed after going through numerous revisions through these evaluation results. 

### 2.5. Application Stage

The evaluation on the application efficacy of the online program was conducted on patients with chronic hepatitis B through a survey that measured hepatitis B related knowledge and self-management compliance after using the online program for three weeks. During this period, the author and research assistants checked and encouraged the use of the online program twice a week. 

The study was performed by subjecting 42 patients with chronic hepatitis B, who were recruited through the outpatient bulletin board at the university hospital and through face-to-face promotion of the online program during their outpatient visits. The author and research assistants initially explained the method of application evaluation by phone or by face-to-face meeting with participants and received their written consent forms. After that, the participants were asked to respond to a questionnaire survey by mail for pretest that measured patients' knowledge and self-management performance level in regard to hepatitis B. For those patients who responded to the questionnaire, the website address was given and allowed them to utilize the online program using their own computers for three weeks in their homes. The author and research assistants of the program phoned them more than twice a week to encourage them to use the program continuously and checked for any presence of difficulty in utilizing the program. For those patients who used the program for three weeks, a postquestionnaire was mailed and asked them for response. The collected data was analyzed through paired *t*-test. 

The hepatitis-B-related knowledge assessment tool is a questionnaire consisting of 28 questions revised and supplemented by Yang [8] based on 20 questions in the chronic hepatitis B knowledge assessment tool developed by Park [27]. The content of the questions includes overall overview, infection route, understanding of liver function test, symptoms, treatment and management, and vaccination, with each question rated as 0 (incorrect) or 1 (correct) point. Higher score means that the knowledge level is higher. The self-management compliance assessment tool is a set of 15 questions revised and supplemented by Yang [8], based on 13 questions of the health behavior assessment tool for patients with hepatitis B developed by Park [27]. The content of the questions includes the diet, activity/rest, the use of extra-medication therapy, drinking, efforts to acquire health information, prevention of transmission, regular checkup/treatment compliance, and observation of complications/adverse drug events. Each question is rated on a 5-point scale with higher points meaning a higher level of self-management compliance. 

## 3. Results

### 3.1. Need Assessment and Analysis

To investigate the needs related to the online program for promoting self-management in patients with chronic hepatitis B, data was collected through literature review, existing websites as well as patients, and their families. The main issues were analyzed from the collected data, and its priority and topic for each issue were set by the expert panel. Finally, other experts reviewed its content validity. 10 main issues emerged in total (Table 1). 

### 3.2. Development

The structure of the online program was created by applying relevant theories such as social cognitive behavior theory [28], self-efficacy theory [29], and problem-solving mediation theory [15] on the basis of self-management model theory for chronic disease patients [14]. An online program for promoting self-management was completed to implement the online system using various strategies that can enhance accessibility and applicability. All content was provided by synchronizing voice, video, and text data to arouse the subject's interest and enhance the educational effect. 

**Figure 2:**
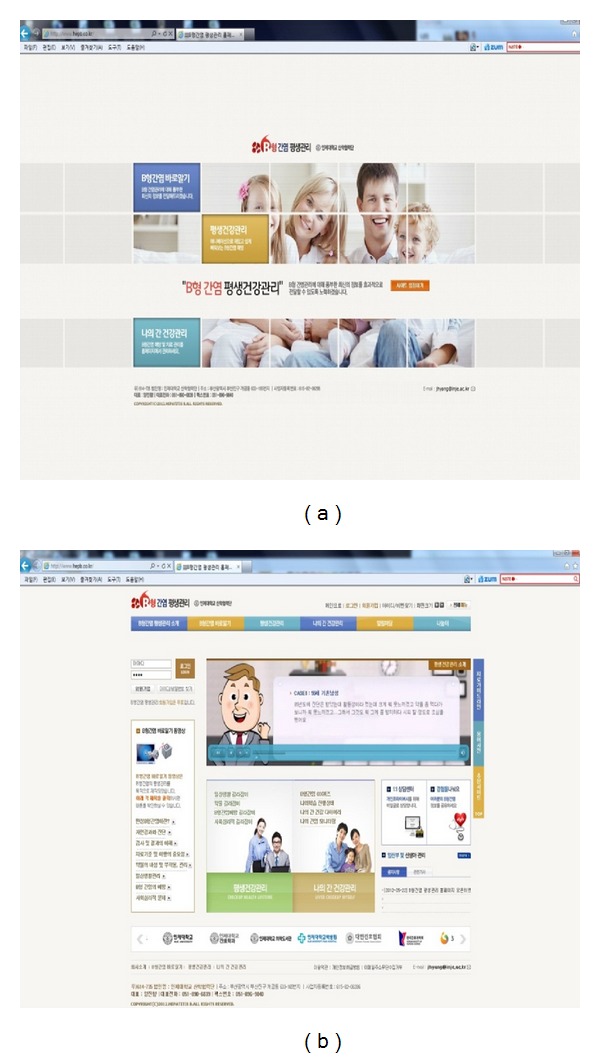
Initial screen and main page.

**Figure 3:**
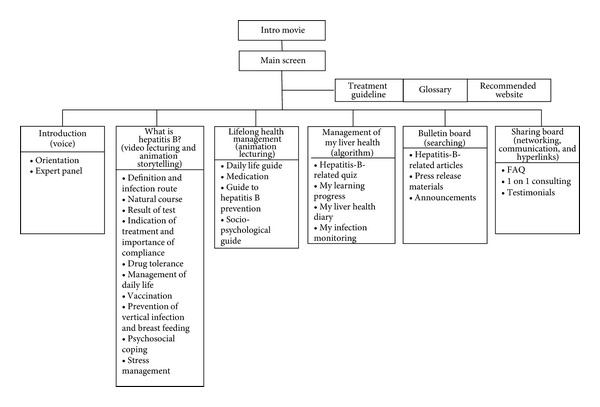
Structure of the online program.

Once you enter the website through the initial screen, it was designed so that the entire menu of the online program can be viewed at a glance (Figure 2). The structure of the online program is consisted of six main menus with submenus and three additional menus (Figure 3). First, “Introduction to Lifelong Management of Hepatitis B” explains why hepatitis B must be managed from the aspect of lifelong health management, and it introduces the overall orientation of the program and the expert panel. Second, in “What is Hepatitis B?,” after writing the actual situation for each issue regarding the 10 main issues with storytelling, the reply to each situation was provided by making educational material using multimedia. The subjects were made to be able to foster their self-management ability through web-based learning utilizing various multimedia materials. Third, “Lifelong Health Management” consists of a guide to four submenus. If subcategories of each guide are examined, daily life guide includes diet, exercise, personal hygiene, and regular checkup; medication guide contains safety precautions upon taking medication, type of medication, and insurance benefit; guide to hepatitis B prevention has infection route, vaccination, vertical transmission prevention, and management during pregnancy and breast feeding; sociopsychological guide covers employment and enlisting in the military, social prejudice, and stress and anxiety control. Fourth, “Management of My Liver Health” consists of four submenus. The subjects can identify their own level of knowledge through a quiz and be provided with feedback on how to cope appropriately through an individual algorithm based on the data they have entered. This data is stored as a database and was made to be utilized in future research. The subjects can check their learning progress. In addition, once one's checkup results, regular checkup date, and taken medication are entered into “My Liver Health Diary,” it reminds him of the next checkup date through a pop-up window each time the website is accessed and re informs him the meaning of the checkup results, importance, and precautions to take in case of complying to the taken medication. In “My Infection Monitoring,” it enables a subject to identify the current stage of one's hepatitis B infection using an individual algorithm and specifically explains what kind of management is needed. Fifth, “Bulletin Board” includes hepatitis-B-related articles, press release materials, and announcements and allows the user to view the latest information and use it for help. Lastly, “Sharing Board” is comprised of three submenus. FAQ was created by preparing replies based on the details frequently asked by patients with chronic hepatitis B and their families. One-on-one consulting was made to raise the subject's level of satisfaction and have them practice health behavior more actively through interactions with experts. Testimonials were designed to allow subjects to interact with other patients and their families, and have them form and vitalize self-help groups while sharing each other's experiences. 

Besides this, three separate menus in addition to the main menu, with contents of much importance and applicability, were designed horizontally on the right hand side. “Treatment Guidelines” suggest standards of beginning treatment, treatment, safety precautions upon treatment, disease monitoring, and standard of finishing treatment. The glossary was designed to instantly provide a description once a patient or his family clicks on a word hyperlinked to a terminology that is difficult to understand. “Recommended Websites” allows patients to conveniently obtain support by providing links to various Korean websites such as hepatitis-B-related societies, government institutions, and social support networks in Korea.

### 3.3. Program Evaluation

The evaluation of the online program established in this study was carried out through an evaluation conducted by an expert panel involved in the treatment and health management of patients with chronic hepatitis B, and user satisfaction of patients with chronic hepatitis B. First, as a result of analyzing the responses from 28 respondents who voluntarily participated in the program, user satisfaction evaluation among outpatients with chronic hepatitis B turned out high in the order of 4.01 points for contents usefulness, 3.85 points for contents relevancy, 3.82 points for design, 3.75 points for system convenience, and 3.68 points for system efficiency out of a perfect score of 5 (Table 2). 

As a result of analyzing the responses from 17 health professionals comprised of physicians and nurses who engage in treatment or management of patients with hepatitis B and three medical informatics experts, purpose had 4.35 points, appropriateness 4.22 points, ease of use 4.14 points, and credibility 4.07 points, which were higher than other criteria; currency had the lowest score of 3.54 points (Table 3). For each item, while purpose, site map, site title, and relevant content of the online program had high scores, the date of last modification, user feedback mechanism, authors of all information, and validation process had relatively low scores. Therefore, these were supplemented in the prototype revision process. 

### 3.4. Application Evaluation

After recruiting 42 patients with hepatitis B through one-on-one promotion at the outpatient clinic and the hospital outpatient bulletin board to evaluate the application of the online program developed in this study, an analysis was conducted on the knowledge and self-management compliance related to hepatitis B to test the effect before and after application (Table 4). As a result of analyzing the differences of the knowledge and self-management compliance between before and after the application of the online program, significant differences were evident. According to analysis by item, in the case of knowledge related to hepatitis B, the correct answer rate of “If my level of liver enzyme is normal, I do not have to do my regular checkup,” and “If my level of liver enzyme reduces to normal levels, I can stop taking medication” significantly increased. In the case of self-management compliance, the level of compliance significantly increased in the items “I do not use nutritional supplements or folk therapy on my own discretion” or “I regularly do my checkup or take my medications as prescribed.” 

## 4. Discussions

As a result of analyzing domestic online systems operated under the topic of “hepatitis B,” a significant number of websites were found to exist to advertise pharmaceutical companies or primary clinics. Most of the information was focused on pathological anatomy and the disease itself, which provided excessively professional or overly superficial information. In cases overseas, there were many websites related to management of hepatitis B, which tend to be more specialized and subdivided than in Korea. However, most of these websites were focused on unidirectional knowledge transfer on disease prevention and management of hepatitis B [7].

It is considered that the management of chronic disease is effective when a program for promoting self-management is carried out in consideration of multidimensional factors, rather than knowledge-oriented education to allow proper health behavior to be internalized from the aspect of lifelong health management, along with specific disease management behavior. To patients with chronic hepatitis B, internalization of continuous disease monitoring, treatment compliance, and proper health behavior are important above everything else [17]. However, precedent studies pointed out the fact that it is difficult to hear what the major concern of patients was because caregivers provide consultation focusing on virus infection to patients with chronic hepatitis [12] and the primary care physician's management of chronic hepatitis B remains at a level of passive disease monitoring since it mainly focuses on test results [30]. It can be seen that the subjects who have applied the online program for promoting self-management developed by this study were helpful in promoting the self-management of patients with chronic hepatitis B by significantly increasing the level of disease-related knowledge and self-management compliance, compared to the instance before application. 

As a consequence of additionally analyzing the subject's disease-related knowledge prior to applying this online program, with regard to questions with incorrect answers at a correct answer rate below 50%, it implied that there is a problem in disease control since patients had an inaccurate knowledge of details. For example, “If my level of liver enzyme is normal, I do not have to do my regular checkup,” and “If my level of liver enzyme goes down to normal levels, I can stop taking medication” is essential knowledge in managing chronic hepatitis B. Many hepatitis B virus carriers' inadequate understanding of the disease resulted in indifferent or inappropriate health-seeking behavior towards their disease management [31]. Additionally, the level of hepatitis-B-related health behavior was significantly higher in subjects with a high level of knowledge on hepatitis B [32]. One qualitative study [33] pointed out that patients with chronic hepatitis B appeared to be unable to hear sufficient explanation on their infectious status and future disease control from caregivers at the initial diagnosis and emphasized that the patients must have adequate guidance and education so that they can have an accurate understanding of their infectious status and better manage their disease in the future. 

 As a result of additionally analyzing the self-management compliance of subjects before the application of the online program, items below the average rating of 3.5 points included “I do not use nutritional supplements or folk therapy on my own discretion,” and “I regularly do my checkup or take my medications as prescribed.” Especially, regular checkups or steady intake of medication are essential items for preventing liver cirrhosis or liver cancer and, ideally, should be a perfect score of 5 points. This lack of compliance, however, is believed to be due to the disease characteristics of chronic hepatitis B, which attack without specific symptoms and progress for a prolonged period [13]. Therefore, it is necessary for nurses to provide guidance to the subjects to have an accurate understanding of their health conditions and disease-related knowledge and take the initiative in practicing health management by adequately applying this program in nursing practice. 

## 5. Conclusions

The ultimate aim of the online program for promoting self-management is to prevent severe liver diseases such as liver cancer beforehand and, furthermore, establish a healthy lifestyle from the aspect of lifelong health management by raising the subject's awareness on the importance of hepatitis B infection management while promoting regular disease monitoring, treatment, and health management compliance. In order to achieve this objective, it is of paramount importance for the patient to understand the characteristic of their disease and promote self-management ability against chronic diseases. 

In the case of the online program, a lifelong health management support system, taking on the approach of fostering problem solving and self-management ability to patients with hepatitis B with various disease progress statuses will be more effective. This online program enhances the effectiveness of self-management with the latest updates through a tailored approach using an individual algorithm without limitation in time and space, web-based interactive learning, consulting with experts, vitalization of self-help group with other patients, and virtual situations and various multimedia materials. Therefore, there is significance in that this research finding allows patients with chronic hepatitis B to engage in proactive and effective health management when applying such online programs for promoting self-management in the community or clinical practice. 

## Figures and Tables

**Table 1:** Main issues of program for promoting self-management of patients with hepatitis B.

Priority	Main issues	Topics
1	“Hepatitis B, is this genetic disease?”	(i) Definition of hepatitis B
		(ii) Infection route of hepatitis B
		(i) Natural course of hepatitis B
2	“I am healthy!”	(ii) Sign and symptom of hepatitis B
		(iii) Importance of disease monitoring
3	“I don't exactly know what GPT is”	Understanding of the results of the test
4	“When do I have to take this medicine?”	(i) Indication of treatment
		(ii) Importance of compliance with medication
5	“Level of liver function test went up again in spite of medication for a long time.”	(i) Drug tolerance and side effect
		(ii) Management of drug tolerance and side effect
6	“Do I have to quit drinking or smoking?”	Management of daily life
7	“Am I a hepatitis B carrier? I got a vaccination against hepatitis B a long time ago”	Vaccine candidates
8	“How do I have to manage not to infect my baby?”	(i) Prevention of vertical infection with hepatitis B
	“Could I perform breast feeding for my baby?”	(ii) Breast feeding
9	“I don't want others notice that I have hepatitis B,”	Psychosocial coping
10	“I'm worrying about getting liver cancer eventually.”	Stress management

**Table 2:** User satisfaction with online program for hepatitis B patients (*n* = 28).

Criteria	Category	Mean score	Mean score of each criteria
System efficiency	Easy to connect	3.73	3.68
	Fast to search information	3.64	

	Easy to contact to operator	3.58	
System convenience	Easy to use the system	3.92	3.75
	Friendly to use	3.75	

Design	Attractive design	3.82	3.82

	Well-arranged information	3.82	
	Up-to-date information	3.79	
	Clear information	3.75	
Content relevancy	Necessary information	3.85	3.85
	Reliable information	3.88	
	Accurate content	4.02	
	Rich content	4.05	
	Detailed content	3.64	

	Content related to me	4.14	
Content usefulness	Useful for my health behavior	3.92	4.01
	Easy to understand	3.96	

**Table 3:** Online program evaluation by expert panel (*n* = 20).

Criteria	Item	Mean score	Mean score of each criteria
	Purpose described	4.58	
Purpose	Intended audience described	4.52	4.35
	Sufficient information provided	4.34	
	Consistent content organization	3.98	

	Appropriate site title	4.15	
	Relevant content for the purpose	4.25	
	Relevant content to intended audience	4.20	
Appropriateness	Relevant content for a subject	4.37	4.22
	Appropriate information presentation	4.28	
	Relevant linkage	4.33	
	Understandable content to intended audience	3.94	

	Accurate information	3.96	
Accuracy	Source of the information	4.28	3.92
	Responsiveness	3.88	
	Validation process described	3.57	

Credibility	Organization behind the site clearly presented	3.78	4.07
	Organization address, phone, email	4.36	

	Site map	4.24	
Ease of use	Structure of the site clearly communicated	4.11	4.14
	Search function	4.08	

Interactivity	Clear user feedback mechanisms	3.52	3.73
	Care of users' feedback	3.95	

	Easy to access	4.08	
Currency	Date of first posting	3.32	3.54
	Date of last modification	3.21	

Authority	Authors of all information	3.56	3.83
	Authors' name and affiliation	4.10	

**Table 4:** Comparison of knowledge and self-management compliance before and after application of the online program (*n* = 42).

Variables	Before		After		*t*	*P*
	Mean	SD	Mean	SD		
Knowledge	17.33	4.13	18.75	3.99	−2.45	.019
Self-management compliance	59.02	7.11	60.59	7.82	−2.30	.027
